# Three new species in the genus *Wilkinsonellus* (Braconidae, Microgastrinae) from the Neotropics, and the first host record for the genus

**DOI:** 10.3897/zookeys.302.4962

**Published:** 2013-05-20

**Authors:** Diana Carolina Arias-Penna, James B. Whitfield, Daniel H. Janzen, Winnie Hallwachs

**Affiliations:** 1 Department of Entomology, 320 Morrill Hall, 505 S. Goodwin Ave., University of Illinois, Urbana, IL 61801, USA; 2 Department of Biology University of Pennsylvania, Philadelphia, PA 19104, USA

**Keywords:** Biodiversity, caterpillars, parasitoid wasps, tropical rain forest lowlands

## Abstract

The genus *Wilkinsonellus* Mason is a poorly sampled but widely distributed tropical genus of Microgastrinae (Braconidae), parasitoid wasps that exclusively attack caterpillars (Lepidoptera). Currently, species of *Wilkinsonellus* have been described only from the Palaeotropics, but the genus was known to occur in the Neotropics. Here we describe the first three species from Central and South America: *Wilkinsonellus alexsmithi* **sp. n.**, *Wilkinsonellus kogui* **sp. n.**,and *Wilkinsonellus panamaensis* **sp. n.** These species descriptions confirm that *Wilkinsonellus* is a Pantropical genus. A dichotomous key for the three new Neotropical species is given. The first recorded host for the genus, *Microthyris prolongalis* (Crambidae), is also reported, for *Wilkinsonellus alexsmithi*.

## Abstract (es)

*Wilkinsonellus* es un género tropical de microgastrinos (Braconidae) que ha sido poco muestreado aunque presenta una distribución amplia. Estas avispas parasitoides únicamente utilizan como hospederos larvas (orugas) de lepidópteros. Hasta el momento, las especies de *Wilkinsonellus* han sido descritas solo a partir de material colectado en la región Paleotropical (trópico del viejo mundo), aunque su presencia en el Neotrópico ya se conocía. En esta publicación se describen las primeras tres especies Neotropicales, dos de Centroamérica y una de Suramérica: *Wilkinsonellus alexsmithi* **sp. n.**, *Wilkinsonellus kogui* **sp. n.**, *Wilkinsonellus panamaensis* **sp. n.** Estas descripciones de especies Neotropicales confirman la distribución Pantropical del género. Una clave taxonómica para la identificación de las tres nuevas especies es propuesta. El primer hospedero para el género, *Microthyris prolongalis* (Crambidae), se reporta para *Wilkinsonellus alexsmithi*.

## Introduction

The genus *Wilkinsonellus* was erected by Mason (1981) to accommodate four Palaeotropical species that Nixon (1965) included in the *Apanteles henicopus* and *Apanteles daira* groups. The former of those two species-groups contained three species, *Apanteles henicopus* (de Saeger 1944) from Kenya and Rwanda, *Apanteles iphitus* (Nixon 1965), and *Apanteles thyone* (Nixon 1965) both from the Philippines, and the *daira*-group is monotypic, with *Apanteles daira* (Nixon 1965) being from Papua New Guinea. In both species-groups, tergite I exhibits a distinctively narrow petiole, which is constricted medially and also with a deeply median groove (Nixon 1965).

After its origin as a recognized genus, the frequency of additional new *Wilkinsonellus* species descriptions has been spasmodic. Eleven years after its acceptance as a new genus, three new species were described from Papua New Guinea and Australia (Austin and Dangerfield 1992); the latter country was added as a new continental record of distribution. In the early twenty-first century, two more species were described, but this time from Northwest Vietnam (Long and van Achterberg 2003). Between 2005-2007, two additional species were described, one from India (Ahmad et al. 2005) and a further one from Vietnam (Long 2007). The most recent descriptions are from Taiwan and Vietnam (Long and van Achterberg 2011), when another four new species were added. Thus, the genus currently contains 15 described species, mainly from the Palaeotropics: Africa, south and Southeast Asia, Indonesia and Australia. The presence of *Wilkinsonellus* in the Neotropics was reported more than a decade ago during the elaboration of a key to Microgastrinae (Whitfield 1997), published in the Manual of the New World Genera of the family Braconidae. However, no Neotropical species were formally described at that time.

Currently, there is no information available about which families of Lepidoptera are used by these wasps as hosts, except that newly reported here. However, they are assumed to be koinobiont endoparasitoids of caterpillars (larvae of Lepidoptera), as are all genera of Microgastrinae. Only one of the previously described species, *Apanteles daira*, has natural history data associated. It was labeled as bred from the plant *Hibiscus*, Malvaceae (Nixon 1965), which is obviously incomplete without an insect host.

Three undetermined species of *Wilkinsonellus* were reported in Kalimantan (Indonesia: Borneo island) during a study that assessed braconid parasitoid wasps diversity after the reforestation of degraded *Imperata* grassland (*Imperata cylindrica*,Poaceae) with *Acacia mangium*,Fabaceae (Maeto et al. 2009). In that study, one undetermined species was reported in matured *Acacia* plantations (aged 5–12 years), and two other unidentified species were found in old secondary logged dipterocarp forests.

The phylogenetic position of the genus within Microgastrinae is unclear. However, some authors have used comparative morphology to suggest a close relationship with *Diolcogaster* Ashmead. Two *Diolcogaster* species-groups have been proposed as close relatives. One of them is the *xanthaspis*-group (Austin and Dangerfield 1992). As with *Wilkinsonellus*, this species-group is characterized by its narrow petiole of tergite I, but the petiole has more or less parallel sides, while that of *Wilkinsonellus* (Nixon 1965) is constricted laterally. The other species-group is the *fasciipennis*-group (Mason 1981), which differs from *xanthaspis* only in that tergites II and III show no delimited median area (Nixon 1965). At the present time, the phylogenetic position of *Wilkinsonellus* within Microgastrinae remains an open question, largely due to little effort having been expended in representing all relevant groups in phylogenetic analyses.

After a brief mention of Dr. Wilkinson’s contribution to the knowledge of Microgastrinae, the first three Neotropical species of *Wilkinsonellus* are described. A Pantropical distribution for the genus is confirmed, along with the first host data for the genus, and we offer a key for the three new species.

### Douglas Shipton Wilkinson (1890–1941)

In 1981, William R. M. Mason named the genus in honor of D. S. Wilkinson, a renowned British entomologist at the Natural History Museum in London - then known as the British Museum (Natural History), who dedicated his entire career to the study of Microgastrinae. Wilkinson was a significant contributor to Microgastrinae taxonomy. He concentrated his efforts in understanding the morphological variability of *Apanteles* not only regionally, but also on a global scale. His vast knowledge of *Apanteles* helped him to design a morphology-based classification (Papp 1976). He proposed six groups; each one was named with arbitrarily chosen letters [A, F, G, S, U, & M] (first developed in Wilkinson 1932). This system of letter-designated groups was adopted, modified and extended from the previous four sections proposed by Marshall (1885) for the British *Apanteles* fauna (Nixon 1965). He was the first European entomologist to recognize the necessity of critically reviewing the classification of the Palaearctic *Apanteles* species. Wilkinson enlisted in the navy during World War II and was killed at sea in 1941, terminating his intention of attaining a world classification of Microgastrinae (Papp 1976, Whitfield et al. 2002).

Wilkinson’s later work on the Palaearctic fauna was published after his death, with the aid of Gilbert Nixon, who became his successor in studying the group (Wilkinson 1945). In this monumental work he re-described 58 European *Apanteles* species in a highly detailed way, and included nomenclatural comments and extensive information on natural history. The detailed critical analysis of host-ranges was due to his proficient collaborator Richard Laurence Edward Ford, who could replicate in the laboratory the conditions of rearing parasitoids and their hosts.

## Methods

Specimens used by this revision were obtained on loan from the following institutions, which are identified in the text by their acronyms:

• Canadian National Collection of Insects (CNC). Ottawa, Canada.

• Entomological collection, Jorge Ignacio Hernández Camacho, Institute of Biological Resources Alexander von Humboldt (IAvH-E). Villa de Leyva, Colombia.

• Inventory Collection of D. H. Janzen and W. Hallwachs (DHJWH) destined for the CNC or National Museum of Natural History, Smithsonian Institution, Washington, D.C.

The specimens from the IAvH–E collection are the result of the project “Insect Survey of a Megadiverse Country Phase I and II: Colombia” conducted from 2002 to 2006. More than 25 natural protected areas managed by the Colombian government were sampled (Arias-Penna 2007). Specimens from the DHJWH collection are the result of “the caterpillar and parasitoid inventory of the Área de Conservación en Guanacaste (ACG) ” Costa Rica (Janzen and Hallwachs 2009, Janzen et al. 2009), a large-scale on-going rearing project. Caterpillars were collected directly in the field and subsequently reared in laboratory conditions. Information about taxonomic identification for caterpillar, host plant and parasitoids as well as data of parasitoid eclosion is available. Each caterpillar is tagged with a voucher code: YY-SRNP-XXXX. The prefix refers to the last two digits of the year that caterpillar was discovered in the field. SRNP stands for Santa Rosa National Park, and the suffix is a unique number assigned within the year. When a parasitoid emerged from its host, the same caterpillar voucher code is assigned, but also a unique DNA wasp voucher code is assigned: DHJPARxxxxxxxx (Janzen and Hallwachs 2009, Janzen et al. 2009).

### Morphology and taxonomic characters

Initial identification to genus level followed the key to the Neotropical microgastrine genera (Whitfield 1997). The original *Wilkinsonellus* species descriptions from Papua New Guinea (Nixon 1965), Australasian Region (Austin and Dangerfield 1992), Vietnam (Long and Achterberg 2003, 2011), India (Ahmad et al. 2005) and China (Zeng et al. 2011) were consulted to confirm that the new species matched the generic aspects of those descriptions. The cuticular sculpturing terminology utilized in this revision follows Harris (1979). Morphological terms for body structures as well as venation are a variation of the Comstock-Needham system that was used by Sharkey and Wharton (1997, fig. 15). Photos were taken with a Leica DFC425 digital microscope camera mounted on a Leica M205 stereomicroscope, (Wetzlar, Germany). The LAS (Leica Application Suite) multifocus module integrated within the Leica microscope was used for taking the pictures. The stack of images at different focus positions was processed with Zerene Stacker version 1.04 (http://zerenesystems.com/cms/stacker).

## Results

### Wilkinsonellus

Mason, 1981

http://species-id.net/wiki/Wilkinsonellus

#### Type species:

*Apanteles iphitus*, Nixon 1965

#### Diagnosis.

**Figure 1.**
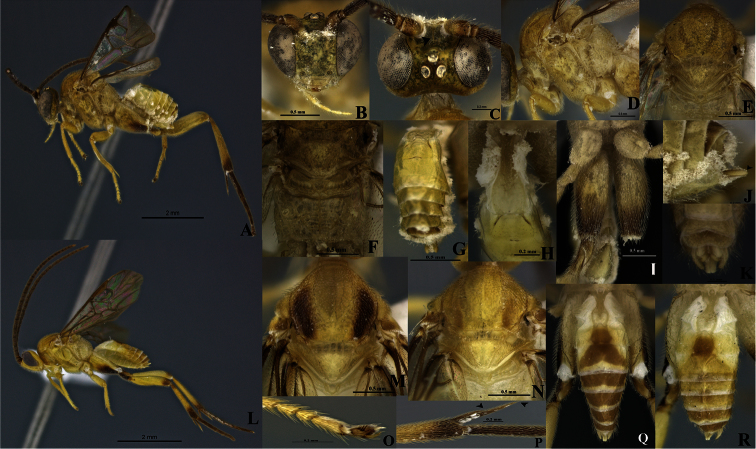
*Wilkinsonellus alexsmithi* Arias-Penna & Whitfield, **A–J** female & **K–R** male **A** Habitus **B–C** Head **B** Frontal view **C** Dorsal view **D–E** Mesosoma **D** Lateral view **E** Dorsal view **F** Scutellum, metanotum & propodeum, dorsal view **G** Last tergites, dorsal view **H** Petiole & Tergites I-II, dorsal view **I** Hindcoxa, ventral view **J** Hypopygium & ovipositor sheaths, lateral view **K–R** Male: **K** Genitalia **L** Habitus **M–N** Mesonotum, dorsal view **O** Claw of hind tarsus **P** Spines of hind tibia **Q–R** Metasoma, dorsal view.

**Figure 2.**
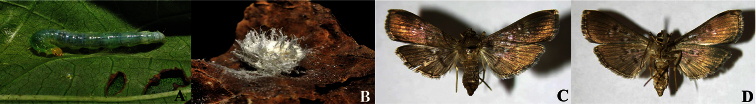
**A** *Microthyris propongalis* (Guenée, 1854) Crambidae: Larva (07-SRNP-41608, Photo: DHJ422561) B Silk and wax cocoon of *Wilkinsonellus alexsmithi* sp. n. (09-SRNP-75793, DHJPAR0039932, photo: DHJ476579) **C–D** Adults of *Microthyris propongalis* **C** Dorsal view **D** Ventral view (06-SRNP-41780 Photos: DHJ349728 & DHJ349729).

**Figure 3.**
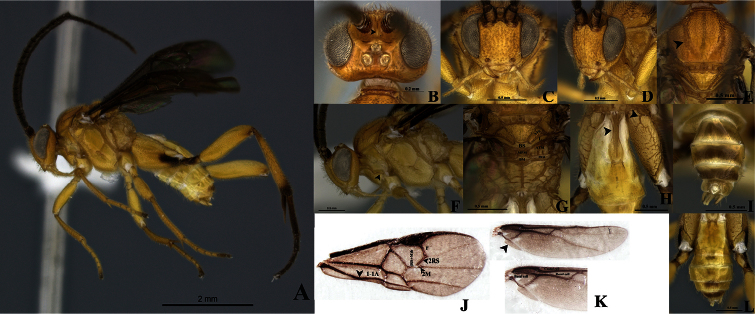
*Wilkinsonellus kogui* Arias-Penna & Whitfield, male. **A** Habitus **B–D** Head **B** Dorsal view **C** Frontal view **D** lateral view **E** Mesosotum, dorsal view **F** Head and mesosoma, lateral view **G** Scutellum, metanotum & propodeum, dorsal view **ATM**= axillary through of metanotum; **ATS**= axillary trough of scutellum; **BM**= Medioposterior band of metanotum; **BS**= medioposterior band of scutellum; **L** = Lunule, **MPM** = Medioanteror pit of metanotum & **PRM** = Posterior rim of metanotum. **H** Tergites I-III & hind coxa, dorsal view **I** Last tergites of metasoma, dorsal view **J** Fore wing veins **K** Hind wing cells **L** Metasoma, dorsal view.

**Figure 4.**
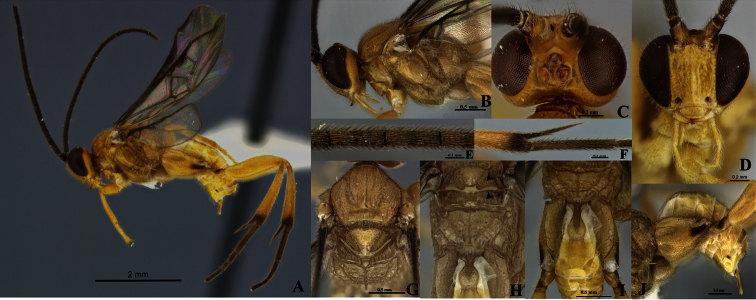
*Wilkinsonellus panamaensis* Arias-Penna & Whitfield, female. **A** Habitus **B** Head & mesosoma, lateral view **C–D** Head **C** Dorsal view **D** Frontal view **E** Antennal flagellomeres **F** Spines on hindtibia **G** Mesosoma, dorsal view **H** Scutellum, metanotum & propodeum, dorsal view **I** Propodeum, Tergites I-V & hindcoxa, dorsal view **J** Metasoma and hindcoxa, lateral view.

*Wilkinsonellus* can be differentiated from other Microgastrinae genera by the combination of the following characters: body coloration largely yellowish (Figs 1A, L; 3A; 4A); propodeum with a median carina, spiracles surrounded by carinae (Figs 1F, 3G, 4H); propleuron with a posterior flange (Figs 1D, 3F, 4B); scutellum sculptured medio-posteriorly and often with subapical carina (Figs 1M–N, 3G, 4H); lunulae of scutellum wide (Figs 1E–F, 3G, 4G–H) (Long and Achterberg 2011), fore wing with second submarginal cell (“areolet”) open distally, thus vein r-m absent (Fig. 3J); vein 1-1A strongly curved, laying very close to posterior margin of the fore wing (Fig. 3J) (Long and Achterberg 2003); tergite I with petiole 4–5 times as long as its apical width, more or less constricted medially and deeply grooved almost to apex (Figs 1H, Q–R; 3H; 4H–I) (Zeng et al. 2011); median longitudinal area of metasomal tergite II slightly raised, usually poorly delimited (Figs 1G–H, Q–R; 3H, L; 4I), tergite II as long as tergite III, both smooth (Figs 2G–H, Q–R; 3H, L; 4I) (Whitfield 1997); hind coxa enlarged (Figs 1A, I, L, Q; 3A, H, L,) rarely short except in *Wilkinsonellus flavicrus* (Long and Achterberg 2011); ovipositor sheaths short (Figs 1A, G, J, 4A, J) (Whitfield 1997).

#### Key to Neotropical *Wilkinsonellus* Mason

**Table:** 

1	Scutellar sulcus with seven carinate foveae (Fig. 4G). Axillary trough of metanotum with complete parallel carinae (Fig. 4H). Eyes and ocelli appearing reddish in preserved specimens (Figs 4A–D)	*Wilkinsonellus panamaensis* sp. n.
–	Scutellar sulcus with five carinate foveae (Figs 1E, M–N; 3E). Axillary trough of metanotum with some incomplete parallel carinae (Figs 1F; 3G). Eyes and ocelli silver in preserved specimens (Figs 1A–C, L; 3A–D, F)	2
2	Fore wing and hind wing infuscate (Figs 3A, J–K)	*Wilkinsonellus kogui* sp. n.
–	Fore wing and hind wing not infuscate (Figs 1A, L)	*Wilkinsonellus alexsmithi* sp. n.

### Descriptions of new species

#### Wilkinsonellus alexsmithi

Arias-Penna & Whitfield sp. n.

urn:lsid:zoobank.org:act:7D233175-9A75-4850-BB55-E314F705F906

http://species-id.net/wiki/Wilkinsonellus_alexsmithi

Figs 1A–R

##### Material examined.

Type material. Holotype, 1 female, COSTA RICA: Alajuela, Area de Conservación Guanacaste, Sector Rincon Rain Forest, Estación Llanura, lat 10.93332, long -85.25331, 135 m, 17.ix.2009, M. Moraga, 09-SRNP-75793, parasitoid voucher DHJPAR0039932. Paratypes: 2 males same data as holotype except for collecting dates and voucher codes as follow: 10.x.2009, 09-SRNP-76107, parasitoid voucher DHJPAR0039933; and 09.x.2009, 09-SRNP-76084, parasitoid voucher DHJPAR0039931. All specimens deposited in DHJWH temporarily, for later transfer to CNC.

##### Diagnosis.

Eyes silver mottled with gray, ocelli silver (Figs 1A–C, L). Curvature of pronotum with a deep groove that has semicircular rugae. Scutellar sulcus with five deep, carinated foveae of heterogeneous size (Figs 1E–F, M–N). Axillary trough of scutellum (ATS) with several parallel carinae that are close to each other (Figs 1F, M–N). Fore wing longer than body length.

Holotype female. Body length 4.56 mm, fore wing length 4.87 mm, hind wing length 3.99 mm

Coloration (Figs 1A–R). General body pale yellow, except posterior half of hind coxa with an infuscated ventral band (Fig. 1I). Flagellum, trochanter, trochantellus, apex of both femur and tibia brown, hind tarsi, and tarsal claws of all legs completely brown. Scape and pedicel yellow-brown. Eyes silver mottled with gray, ocelli silver (Figs 1A–C, L). Membrane and microtrichiae of both fore and hind wings light brown (Figs 1A, L).

Head (Figs 1B–C). Scape longer than wide (0.26:0.17 mm); pedicel wider than long (0.12:0.10 mm), first antennal flagellomeres not sub-equal in length (0.30:0.36:0.34 mm). Antennal scrobes deep, smooth, far above middle level of eyes (Fig. 1B), carinated dorsally (Fig. 1C); in frontal view, medial area between antennal scrobes with a sharp, short projection carrot-shaped (Fig. 1B), antennal scrobes in contact with inner eye margin (Fig. 1B). Face with small, sparse and homogeneous punctures, face with a median-longitudinal carina running from antennal scrobes to clypeus, fronto-clypeal suture absent (Fig. 1B). Distance between each anterior tentorial pit and closest inner compound eye margin equal to diameter of a tentorial pit (0.06:0.06 mm); anterior tentorial pits far away from each other (0.30 mm) (Fig. 1B). Mandible with two teeth, inferior tooth thinner, longer than superior. Maxillary palps longer than labial palps (Fig. 1B). Distance between a posterior ocellus and adjacent eye margin sub-equal in length equal to diameter of lateral ocellus (0.10:0.10 mm), distance between lateral ocelli shorter than diameter of lateral ocellus (0.06:0.10 mm) (Fig. 1C). Vertex narrow with small, sparse punctuations, but medially smooth and concave (Fig. 1C).

Mesosoma (Fig. 1A, D–F, L–N). Mesosoma dorsoventrally convex (Figs 1A, D). Pronotum shiny, smooth; curvature of pronotum with a deep groove that has semicircular rugae. Mesopleuron convex, extended, smooth except margins lateral and ventro-lateral that form a L-shaped area that possesses small, homogeneous punctuations (Fig. 1D), mesopleuron with a deep dent just above L-shaped area, dent with elongated foveae bordering the L-shape area, mesosternum slightly flat with distinctive groove of deep, homogeneous foveae. Metepisternum and metepimeron separated by a groove with several deep foveae throughout (Fig. 1D), metepisternum narrower than metepimeron, metepisternum just above hindcoxa outlined by a wide and flat carina, and apical half with several short cariane. Mesoscutum as wide as head with small and homogenous punctures. Notauli clearly impressed, broad, but not reaching the transscutal articulation (Fig. 1E). Scutellar sulcus with five deep, carinated foveae of heterogeneous size (Figs 1E–F, M–N). Scutellum shiny, almost smooth with sparse, fine punctures and surrounded by a strong carina (Figs 1E–F, M–N). ATS with several parallel carinae which are close to each other (Figs 1E, N). Axillary trough of metanotum (ATM) with a few, incomplete parallel carinae, only present basally (Figs 1E, N). Lunule of scutellum (L) and medioposterior band of scutellum (BS) smooth and shiny. Medioposterior band of metanotum (BM) short and crossed by a carina aligned with the median longitudinal carina of propodeum (Fig. 1F). Medioanteror pit of metanotum (MPM) hexagonal, and delimited by a strong carina (Fig. 1F). Posterior rim of metanotum (PRM) thin and smooth (Fig. 1F). Propodeum with a complete median-longitudinal carina dividing the propodeum in two halves, plus one divergent carina at each half of propodeum, area between carinae basally shorter than apically, divergent carinae crossed by semicircular carinae (Fig. 1F).

Wings (Figs 1A, L). Fore wing with vein r straight (0.30 mm) arising just beyond middle of pterostigma; vein 2RS as long as r (0.30:0.30 mm), but longer than 2M and (RS+M) b veins (0.30:0.15:0.20 mm). Hind wing with vannal lobe reduced, slightly convex; edge with sparse setae throughout. Costal and basal cell infuscate.

Legs (Figs 1A, I, L, O–R). Hind coxa surpassing apex of tergite III (Figs 1A, L, Q–R), outer dorsal surface of hind coxa delimiting an area surrounded by a strong longitudinal carina running from base to apex, but last third apically the carina turns inward (Fig. 1Q); that area with rugulose punctuations and with an extra strong basal carina inclined and reaching only the first third basally; hind tibia with outer spur half as long as inner spur (0.34:0.66 mm); inner spur more than half as long as hind basitarsus (0.66:0.90 mm) (Fig. 1P); hind tibia and hind tarsi both with spines throughout, hind tarsal claw with a short comb (Fig. 1O).

Metasoma (Figs 1G–H, J–K, Q–R). Petiole of tergite I narrow (Figs 1H, Q–R), length 0.56 mm, distinctly constricted at anterior half (minimum width 0.09 mm), but subapically wider (maximum width 0.25 mm) and with a few sculpturations, petiole with a deep groove extending more of two thirds tergite I length; hypopygium not protruding at apex of metasoma (Figs 1A, J); hypopygium plate with truncate apex (Fig. 1J), ovipositor sheath length 0.20 mm, glabrous, slightly protruding apex of metasoma (Fig. 1J).

Males (Figs 1K–R). Males differ in coloration from the female: lateral mesonotal lobes pale or dark brown (Figs 1M–N). Tergite II with a brown median area which is longer than wide (Figs 1Q–R); tergite III with a brown (Fig. 1Q) or yellow-brown area (Fig. 1R) anteriorly narrower than posteriorly; tergites IV brown but subapically with a thin transversal yellow apical band (Figs 1Q–R). The infuscate areas on hind legs are darker than in females (Fig. 1L). Antennae length = 5.0-5.2 mm, body length = 4.2-4.5 mm. Last antennal segment gradually narrowing at the apex. Tergite I, minimum width = 0.10 mm, maximum width = 0.22 mm, total length = 0.60-0.70 mm.

##### Etymology.

This species is named in honor of Dr. M. Alex Smith of the University of Guelph, Canada, in recognition of his decade of deep intellectual, laboratory and logistic support for the DNA barcoding of the parasitoid wasps and flies of ACG.

##### Distribution.

The species is only known from the original rain forest collection site, Sector Rincon Rain Forest, in Área de Conservación Guanacaste in northwestern Costa Rica. In 1999, ACG was inscribed as a UNESCO World Heritage site containing the best-preserved and regenerating dry forest habitats from Central America to northern Mexico.

##### Host.

*Wilkinsonellus alexsmithi* has been reared from the leaf-roller *Microthyris prolongalis*,Crambidae (Figs 2A, C–D) three times, while feeding on the rain forest leaves of *Ipomoea phillomega* or sweet potatoes *Ipomoea batatas* (Convolvulaceae) (http://janzen.bio.upenn.edu/caterpillars/database.lasso). The larva of *Microthyris prolongalis* lives inside of the leaf roll that it constructs, eating leaf tissue there. It is therefore likely that oviposition takes place through the leaf into the moth larva. The wasp cocoon (Fig. 2B) is lightly silked to the inner wall of the leaf roll and the larva dies at about the time that the wasp larva exits the cadaver.

##### Comments.

The last three antennal segments are missing from the holotype. *Wilkinsonellus alexsmithi* is a parasitoid of a crambid leaf roller larva, *Microthyris prolongalis* (Crambidae). In ACG, this moth larva feeds only on Convolvulaceae (410 rearing records, Janzen & Hallwachs 2009). Within the subfamily Microgastrinae besides *Wilkinsonellus*, members of two other genera, *Apanteles* and *Diolcogaster*, are parasitoids only on this species of moth. The taxonomic range of insect parasitoids that use *Microthyris prolongalis* as a host entails two insect orders, Hymenoptera and Diptera. Within Hymenoptera the chalcidoid family Encyrtidae (genus not reported), and two additional subfamilies of Braconidae, Orgilinae (*Stantonia*) and Agathidinae (*Alabagrus maya*) were reported; for the Diptera parasitoids, two genera of Tachinidae, *Actia* and *Argyrophylax* also parasitize this caterpillar (Janzen and Hallwachs 2009).

#### Wilkinsonellus kogui

Arias-Penna & Whitfield sp. n.

urn:lsid:zoobank.org:act:FC7BA3D2-3503-4DD9-B9F7-AADC34BF13CB

http://species-id.net/wiki/Wilkinsonellus_kogui

Figs 3A–L

##### Material examined.

Type material. Holotype. Male, COLOMBIA Magdalena, PNN [Parque Nacional Natural] Tayrona Pueblito, lat 11.33333, long -74.03333, 225m, Malaise, 03-22.i.2001, R. Henriquez leg. M.1212. Paratype. 1 Male, COLOMBIA Chocó, PNN [Parque Nacional Natural] Utría, Cocalito, 6°1’N, 77°20’W, 20m, Malaise, 26.xii.2000–01.ii.2001, J. Pérez, Leg. M.1342. Holotype and paratype deposited in IAvH-E.

##### Diagnosis.

Eyes and ocelli silver (Figs 3A–D, F). Scutellar sulcus with five deep, heterogeneous and carinated foveae (Fig. 3E). Axillary trough of metanotum with a few striated grooves defined at least posteriorly (Fig. 3G). Body longer than fore wing (Fig. 3A).

Holotype male. Body length 4.30 (4.30-4.55 mm), fore wing length 4.15mm, hind wing length 3.59 mm.

Coloration (Figs 3A–L). General body dark yellow; all legs yellow, except hind leg: coxa infuscated at the apex forming a ventral, wide brown band; apex of trochanter, and trochantellus, base of tibia and tarsi brown (Fig. 3A). Scape and pedicel brown both with thin apical yellow ring. Flagellum dark brown. Eyes and ocelli silver (Figs 3A–D, F.) Tergite IV and beyond mostly brown, but subapically and subbasally with a transverse yellow band (Figs 3I, L). Membrane and microtrichiae of fore and hind wing infusctate (Figs 3J–K).

Head (Figs 3A–D). Scape slightly longer than wide (0.20:0.18 mm); pedicel wider than long (0.12:0.08 mm); first three flagellomeres subequal in length (0.32:0.30:0.34). Antennal scrobes smooth, dorsally carinate (Fig. 3B), positioned far above middle level of eyes (Figs 3C–D); median part between antennal scrobes with a short carina (Fig. 3B). Face with sparse, homogeneous and medium-sized punctures, interspaces wavy; face with a median-longitudinal carina running from antennal scrobes to fronto-clypeal suture (Fig. 3D). Distance between each anterior tentorial pit and closest inner compound eye margin longer than diameter of tentorial pit (0.10:0.06 mm) (Figs 3C–D); anterior tentorial pits far away from each other (0.26 mm) (Fig. 3C). Fronto-clypeal suture absent (Figs 3C–D). Mandible with two teeth, inferior tooth thinner and longer than superior (Fig. 3C). Suture malar present (Fig. 3D). Maxillary palps longer than labial palps (Fig. 3C). Distance between lateral ocellus and adjacent compound eye margin longer than diameter of lateral ocellus (0.11:0.08 mm) (Fig. 3B), distance between lateral ocelli equal to diameter of lateral ocellus (0.08:0.08 mm) (Fig. 3B). Vertex medially smooth, but laterally with some sparse and small punctuations. Occiput slightly concave with a median short grove basally.

Mesosoma (Figs 3A, E–G). Mesosoma dorsoventrally convex (Figs 3A, F). Pronotum shiny, smooth, but curvature of pronotum with elongate areolae. Mesopleuron shiny, smooth medially, but margins lateral and ventro-lateral forming a L-shaped area which small, dense and homogeneous sculptures (Fig. 3F); mesopleuron just above of L-shape area with a dent with some large wave-like sculpturing. Mesosternum slightly flat with a deep row of deep foveae. Metepisternum and metepimeron outlined by a groove with several deep foveae throughout (Fig. 3F), metepisternum inverted triangular, smooth and narrower than metepimeron (Fig. 3F), apical margin metepisternum (above hindcoxa) delimited by a wide, flat carina (Fig. 3F). Mesoscutum as wide as head with small, dense, and homogenous sculptures. Notauli clearly impressed, but not reaching the transscutal articulation (Fig. 3E). Scutellar sulcus heterogeneous, with five deep, heterogeneous and carinated foveae (Figs 3E, G). Scutellum shiny, medially smooth, but with sparse fine punctures and surrounded by carina (Figs 3E, G). Axillary trough of scutellum with several homogeneous striated grooves (Fig. 3G). Axillary trough of metanotum with a few striated grooves defined at least posteriorly (Fig. 3G). Medioposterior band of scutellum slightly wider than lunule of scutellum both smooth and shiny (Fig. 3G). Medioposterior band of metanotum hexagonal and crossed by a median carina aligned with the median longitudinal carina of propodeum (Fig. 3G). Medioanteror pit of metanotum pentagonal-shape surrounded by carina (Fig. 3G). Posterior rim of metanotum thin, wavy and smooth (Fig. 3G). Propodeum with a complete median-longitudinal carina dividing in two halves, each half with one divergent carina wider as they go away from propodeal foramen, space among all carinae intercepted by transverse semicircular carinae (Fig. 3G).

Wings (Figs 3A, J–K). Fore wing with vein r length 0.26 mm slightly curved, arising beyond middle of pterostigma, arising just beyond middle of stigma (Fig. 3J); vein 2RS as same length as r (0.26:0.26 mm),but 2RS vein longer than 2M and (Rs+M) b veins (0.26:0.10:0.20 mm) (Fig. 3J). Hind wing with vannal lobe reduced, slightly convex; edge with sparse setae throughout (Fig. 3K). Costal and basal cells infuscate (Fig. 3K).

Legs (Figs 3A, H, L). Hind coxa very long, reaching apex of tergite III (Fig. 3H), outer dorsal surface of hind coxa delimited by a strong carina, area coarsely rugulose and with a short, strong basal carina (Fig. 3H); hind tibia with outer spur more than half as long as inner spur (0.40:0.66 mm), inner more than half as long as hind basitarsus (0.66:0.88 mm) (Fig. 3A), hindtibia and tarsi with spines throughout.

Metasoma (Figs 3A, H–I, L). Petiole of tergite I narrow (Fig. 2H), length 0.70 mm, distinctly constricted at upper middle (minimum width 0.09 mm) and wider subapically (maximum width 0.20 mm) with sculpturations, petiole with a deep groove extending more of two third of the tergite I length (Fig. 3H). Male genitalia externally visible (Fig. 3I).

Female. Unknown

##### Etymology.

From Kogui = jaguar in the Kogui language. The Kogui are indigenous in the Colombia Caribbean coast at the foot of the Sierra Nevada de Santa Marta, the highest coastal mountains in the world and not directly attached to the Andean mountain range.

##### Distribution.

Colombia, from PNN Tayrona and PNN Utría, both being marine ecosystems protected by the Colombian government and belonging to the National Natural systems. Tayrona is located on the Caribbean coast in Magdalena Department, whereas Utría is located on Colombia’s Pacific coast, in Chocó Department.

##### Host.

Unknown

##### Comments.

Holotype lacks the last antennal flagellomeres. The specimens from Utría with antennae length = 4.8 mm, body length 4.3 mm. Last antennal flagellomere length = 0.35 mm, penultimate flagellomere antennae length = 0.30 mm. Male from Chocó shows hind legs with the same pattern of coloration but darker and Tergite VI and beyond with brown spots (Fig. 3L).

#### Wilkinsonellus panamaensis

Arias-Penna & Whitfield sp. n.

urn:lsid:zoobank.org:act:A3274FC6-02B2-4292-9A5B-B37D142516D1

http://species-id.net/wiki/Wilkinsonellus_panamaensis

Figs 4A–J

##### Material examined.

Type material. Holotype. Female, PANAMAProvincia Panamá, Distrito de Panamá, Las Cumbres. Malaise 20.i-02.ii.1982. M. Wolda. DNA Voucher CNCHYM03459. Specimen deposited in CNC.

##### Diagnosis.

Eyes and ocelli appearing reddish in preserved specimens (Figs 4A–D). Metasoma curve (Fig. 4J). Fore wing longer than body length. Vein 2M as long as (Rs+M) b. Scutellar sulcus with seven carinated foveae heterogeneous in size (Fig. 4G). Axillary trough of scutellum and axillary trough of metanotum both with complete parallel carinae (Fig. 4H).

Holotype female. Body length 4.18 mm, fore wing length 4.44 mm, hind wing length 3.43 mm.

Coloration (Figs 4A–J). General body dark yellow, except hind leg infuscated at the base and apex of tibia; hind coxa, trochanter and trochantellus with a narrow dorsal band (Fig. 4J). Hind tarsi completely brown (Fig. 4A). Scape half basal brown and half apical yellow (Fig. 4D). Pedicel brown with yellow apical ring (Fig. 4D). Flagellum brown (Fig. 4E), ocelli and eyes appearing reddish in preserved specimens (Figs 4A–D).

Head (Figs 4A–D). Antenna longer than body (4.44:4.18 mm); scape longer than wide (0.22:0.16 mm); pedicel wider than large (0.12:0.10 mm); first antennal flagellomeres sub-equal in length (0.34:0.32:0.32 mm); penultimate flagellomere as same length than apical segment (0.11:0.11mm); but with flat, abruptly acute in apex. Antennal scrobes smooth, far above middle level of eyes (Fig. 4D) and carinate dorsally (Fig. 4C), median part between antennal scrobes with a short carina (Fig. 4C). Face with small, sparse and homogeneous punctures, face with a median-longitudinal carina running from antennal scrobes to clypeus (Fig. 4D), fronto-clypeal suture absent (Fig. 4D). Distance between an anterior tentorial pit and inner compound eye margin equal to diameter of a tentorial pit (0.06:0.07 mm); anterior tentorial pits far away from each other (0.24 mm) (Fig. 4D). Mandible with two teeth, inferior tooth thinner and longer than superior. Maxillary palps longer than labial palps (Fig. 4D). Distance between lateral ocellus and adjacent compound eye margin sub-equal in length to the diameter of the lateral ocellus (0.09:0.10 mm), distance between lateral ocelli shorter than diameter of lateral ocellus (0.06:0.10 mm) (Fig. 4D). Vertex narrow, medially with a smooth area, but laterally with small and sparse punctuations. Occiput slightly concaved with a short grove medially.

Mesosoma (Figs 4A–B, G–H). Mesosoma dorsoventrally convex (Figs 4A–B). Pronotum shiny, smooth, but curvature of pronotum with a deep grove. Mesopleuron convex, extended smooth except margins lateral and ventro-lateral that form a L-shaped region that possesses small, dense and homogeneous punctuations (Fig. 4B); mesopleuron with a deep dent just above of L-shaped area, demarcating the border of the area with elongate foveae (Fig. 4B). Mesosternum slightly flat with a median row of foveae. Metepisternum and metepimeron separated by a groove with several deep foveae throughout (Fig. 4B), metepisternum smooth and narrower than metepimeron, apical margin metepisternum just above hindcoxa outlined by a wide, flat carina (Fig. 4B). Mesoscutum as wide as head, with small, sparse and homogenous punctures. Notauli clearly impressed, but not reaching the transscutal articulation (Fig. 4G). Scutellar sulcus with seven deep, carinated foveae of heterogeneous size (Fig. 4G). Scutellum shiny with fine, sparse punctures and delimited by carina. Axillary trough of scutellum and axillary trough of metanotum both with vertical parallel carinae (Fig. 4H); space among ATM carinae wider than ATS carinae (Fig. 4H). Lunule of scutellum and medioposterior band of scutellum smooth and shiny (Fig. 4H). Medioposterior band of metanotum and medioanterior pit of metanotum forming a pentagonal-shaped delimited by carinae (Fig. 4H). Posterior rim of metanotum thin and smooth (Fig. 4H). Propodeum with a complete median-longitudinal carina dividing the propodeum in two halves, each half with one additional carina that does not branch basally at the same point than median-longitudinal carina (Figs 4H–I); space between median and an additional carina wider as they become more distant from propodeal foramen (Figs 4H–I), and all three carinae crossed by transverse semicircular carinae, although apically less transverse carinae than basally.

Wings (Fig. 4A). Fore wing with vein r straight (0.27 mm), arising just beyond middle of stigma; vein 2RS longer than 2M and (Rs+M) b veins (0.24:0.10:0.10 mm). Hind wing with vannal lobe reduced, slightly convex; edge with sparse setae throughout. Costal and basal cell infuscate.

Legs (Figs 4A, F, I–J). Hind coxa reaching apex of tergite III (Fig. 4I), outer dorsal surface of hind coxa with an area delimited by a strong longitudinal carina running from base to apex, but last third apically the carina turns inward (Fig. 4I); area surrounded by the carina with rugulose punctuations that are more visible in dorsal view, and with an additional basal carina which splits and runs only the first third basally (Fig. 4I); hind tibia with outer spur half as long as inner spur (0.36:0.72 mm); inner spur more than half as long as hind basitarsus (0.72:0.92 mm) (Fig. 4F); outer dorsal side of hind tibia moderately spinose (Fig. 4F).

Metasoma (Figs 4A, I–J). Petiole of tergite I narrow (Fig. 4I), length 0.52 mm, distinctly constricted at anterior half (minimum width 0.10 mm), but subapically wider (maximum width 0.26 mm); petiole with a deep groove extending more of two thirds tergite I length (Fig. 4I); hypopygium not protruding beyond apex of metasoma (Fig. 4J), hypopygium plate with truncate apex (Fig. 4J); ovipositor sheaths length 0.36mm, glabrous, with apex rounded protruding apex of metasoma (Fig. 4A, J)

Males. Unknown.

##### Etymology.

The name is based on the country of Panamá, where the holotype was collected.

##### Distribution.

The species is only known from the original collecting site in Panamá.

##### Host.

Unknown.

## Conclusions

Neotropical *Wilkinsonellus* range from 4.0 to 4.8 mm in length, excluding antennae, and all specimens were collected in lowland tropical rain forest 500 m.a.s.l. or lower in elevation. Palaeotropical *Wilkinsonellus* range from 2.5 to 4.8 mm in length, and occur at altitudes up to 1700 m.a.s.l.

*Wilkinsonellus* has not beenthe only genus within Microgastrinae that was initially believed to be confined to a specific zoogeographical region. This is also true for *Austrocotesia* Austin and Dangerfield and *Parapanteles* Ashmead, each of which has turned out to have a much wider distribution. *Austrocotesia* was erected as a new genus in 1992. In that time, it was considered restricted to Papua New Guinea and the adjacent Australian region of North Queensland (Austin and Dangerfield 1992). However, the first two species from South America–Colombia and Ecuador– were described thirteen years later (Valerio and Whitfield 2005). Equally, *Parapanteles* wasoriginally recorded only from the Australian and American continents. Ashmead proposed the genus in 1900. However, after little more than a century, *Parapanteles* was reported in South Africa –Western Cape province, Cederberg (Valerio et al. 2005). *Austrocotesia* was also reported from the Neotropics at the same time as *Wilkinsonellus* in a chapter on Microgastrinae (Whitfield 1997) included in the Manual of the New World genera of the family Braconidae (Hymenoptera). Another genus mentioned in that manual was the Afrotropical genus *Beyarslania*,formerly known as *Xenogaster* (Koçak and Kemal 2009). However, those undescribed Neotropical *Beyarslania* species possibly belong to *Mariapanteles*,a genus recently erected (Whitfield et al. 2012). Thus, *Beyarslania* is still restricted to the Afrotropical region. In brief, all the new records point suggest that *Wilkinsonellus* as well as *Austrocotesia* and *Parapanteles* have a more extensively Gondwanan distribution.

The new distribution of *Wilkinsonellus* has been discovered thanks to large-scale rearing projects as well as arthropod diversity surveys undertaken in recent decades in the Neotropical region. The importance of rearing projects lies in the fact the associations across more than two trophic levels are possible. The identification of parasitized larval hosts implies the use of external morphological characteristics present on the larvae combined with the food plant and microlocation, because the caterpillar host is often destroyed when the larval parasitoid emerges. In contrast, specimens collected by malaise trap contribute mainly to inventory of biological diversity, except that most ecological information is lacking. Notwithstanding the current efforts, the extreme richness of the Neotropics means that both taxonomic and biological records continue to be highly incomplete (Whitfield et al. 2002) and more studies are considered necessary in order to improve understanding of the distribution patterns of the Neotropical Microgastrinae fauna. This situation also applies to other critical areas in the planet (i.e., Wallacea, New Guinea, Solomon Islands) that are still unexplored; consequently the understanding of the global distribution patterns remains incomplete.

## Supplementary Material

XML Treatment for Wilkinsonellus

XML Treatment for Wilkinsonellus alexsmithi

XML Treatment for Wilkinsonellus kogui

XML Treatment for Wilkinsonellus panamaensis
